# Mechanism of Action of Shenerjiangzhi Formulation on Hyperlipidemia Induced by Consumption of a High-Fat Diet in Rats Using Network Pharmacology and Analyses of the Gut Microbiota

**DOI:** 10.3389/fphar.2022.745074

**Published:** 2022-04-05

**Authors:** Shuang Zhang, Yu Wang, Fang Lu, Shadi A. D. Mohammed, Hanxing Liu, Song Ding, Shu-min Liu

**Affiliations:** Institute of Traditional Chinese Medicine, Heilongjiang University of Chinese Medicine, Harbin, China

**Keywords:** network pharmacology, gut microbiota, hyperlipidemia, 16S rRNA gene, high-fat diet

## Abstract

Shenerjiangzhi formulation (SEJZ) is a new traditional Chinese medicine formulation (patent number: CN110680850A). SEJZ contains *Eleutherococcus senticosus* (*Rupr.* and *Maxim.*), *Maxim* (Araliaceae; *E. senticosus* radix and rhizome), *Lonicera japonica* Thunb (Caprifoliaceae; *Lonicera japonica* branch, stem), *Crataegus pinnatifida* Bunge (Rosaceae; Crataegus pinnatifida fruit), and *Auricularia auricula*. SEJZ has been designed to treat hyperlipidemia. Despite the therapeutic benefits of SEJZ, its underlying mechanism of action is not known. We explored the efficacy of SEJZ against hyperlipidemia by integrating network pharmacology and 16S rRNA gene sequencing and elucidated its mechanism of action. First, SEJZ targets were found through the Traditional Chinese Medicine Systems Pharmacology Database and Analysis Platform and from the literature. Hyperlipidemia-related therapeutic targets were obtained from GeneCards, Online Mendelian Inheritance in Man, and DrugBank databases. Then, Search Tool for the Retrieval of Interacting Genes/Proteins and Cytoscape were applied for the analyses and construction of a protein–protein interaction (PPI) network. The Kyoto Encyclopedia of Genes and Genomes database was employed to identify signaling pathways that were enriched. Second, the therapeutic effects of SEJZ against hyperlipidemia induced by consumption of a high-fat diet in rats were evaluated by measuring body weight changes and biochemical tests. SEJZ treatment was found to alleviate obesity and hyperlipidemia in rats. Finally, 16S rRNA gene sequencing showed that SEJZ could significantly increase the abundance of short-chain fatty acid-producing bacteria, restore the intestinal barrier, and maintain intestinal-flora homeostasis. Using PICRUSt2, six metabolic pathways were found to be consistent with the results of network pharmacology: “African trypanosomiasis”, “amoebiasis”, “arginine and proline metabolism”, “calcium signaling pathway”, “NOD-like receptor signaling pathway”, and “tryptophan metabolism”. These pathways might represent how SEJZ works against hyperlipidemia. Moreover, the “African trypanosomiasis pathway” had the highest association with core genes. These results aid understanding of how SEJZ works against dyslipidemia and provide a reference for further studies.

## Introduction

Hyperlipidemia is a common disorder of lipid metabolism. It is one of the leading risk factors for cardiovascular disease, atherosclerosis, and other metabolic disorders. Hyperlipidemia is often manifested by obesity (Hu et al., 2019). Hyperlipidemia is characterized by abnormal levels of lipids: an increased level of total cholesterol (TC), triglycerides (TG), and low-density lipoprotein (LDL) and a decrease in the high-density lipoprotein (HDL) level. This dysregulation of lipid levels is usually caused by multiple factors, including genetic factors and an unbalanced diet. It has been reported that 41.2% of individuals aged 40–64 years have abnormal lipid levels in blood, which aggravates the risk of hyperlipidemia greatly (Pencina et al., 2014).

Lipid-lowering medication has undergone a revolutionary development since the introduction of statins (Hirakawa and Shimokawa, 2001). However, severe side effects (e.g., muscle injury) have hindered further clinical application (Rosenson et al., 2017; White et al., 2020). Therefore, more attention has been paid to the research and development of drugs with lower toxicity and greater efficacy, especially those derived from natural products. Studies have demonstrated that traditional Chinese medicine (TCM) formulations can be utilized to regulate the homeostasis of intestinal flora and, thus, have a role in the treatment of disease (Li Y. et al., 2020). After oral administration of a TCM formulation, the active ingredients interact with intestinal flora, reshape the structure of intestinal microbial communities, and play a part in preventing diseases (Yang et al., 2017b; Li Y. et al., 2020).

The “second genome” of the human body is the gut microbiota (GM). The latter can extract energy from indigestible dietary components to promote host metabolism and play a part in synthesizing vitamins and producing important bioactive metabolites, such as short-chain fatty acids (SCFAs) (Cani et al., 2008; Norris et al., 2016). Furthermore, disorders of lipid metabolism are related to an imbalance in intestinal microflora (Han et al., 2019). In patients with hyperlipidemia, high expression of bacteria of the phylum Firmicutes and genera *Lachnospira* and *Haemophilus* and low expression of bacteria of the phylum Bacteroidetes and genera *Ruminococcus*, *Blautia*, and *Roseburia* are found, which hinder SCFA production. Increased intestinal permeability, which leads to chronic inflammation and interferes with cholesterol transport, thereby increases susceptibility to certain metabolic disorders, such as diabetes mellitus and obesity (Granado-Serrano et al., 2019).

Shenerjiangzhi formulation (SEJZ; patent number: CN110680850A) consists of *Eleutherococcus senticosus* (*Rupr.* and *Maxim.*) *Maxim* (Araliaceae; *E. senticosus* radix and rhizome), *Lonicera japonica* Thunb (Caprifoliaceae; *Lonicera japonica* branch, stem), *Crataegus pinnatifida* Bunge (Rosaceae; Crataegus pinnatifida fruit), and *Auricularia auricula*. SEJZ has been employed to treat hyperlipidemia caused by stasis due to excessive accumulation of heat toxins, as well as weakness of the spleen and stomach. The polysaccharides in *E. senticosus* (*Rupr.* and *Maxim.*) and *Maxim* (Araliaceae; *E. senticosus* radix and rhizome) can improve glucose utilization and regulate blood levels of lipids (Zhang et al., 2021). The protocatechuic acid and saponins in *E. senticosus* (*Rupr.* and *Maxim.*) and *Maxim.* (Araliaceae; *E. senticosus* radix and rhizome) are closely correlated with lipid levels in the blood, and triterpenoid saponins have been reported to have optimal activity against hyperlipidemia (Shi et al., 2020; Li X. J. et al., 2021). Various trace elements (e.g., zinc) are contained in *L. japonica* Thunb (Caprifoliaceae; *Lonicera japonica* branch, stem) and *C. pinnatifida* Bunge (Rosaceae; Crataegus pinnatifida fruit) have been demonstrated to prevent and treat hyperlipidemia (Xu and Xu, 2009). *C. pinnatifida* Bunge (Rosaceae; Crataegus pinnatifida fruit) is the most commonly used single botanical drug to treat hyperlipidemia (Chu et al., 2015). Apigenin (Wu et al., 2021), kaempferol (Chang et al., 2011) (Chen et al., 2016), and quercetin (Li H. et al., 2021) are flavonoids and are the main active components of four botanical drugs in SEJZ. They play an important part in improving lipid metabolism disorders and lipid accumulation. Several studies have demonstrated that flavonoids can inhibit traditional inflammatory signaling pathways to lower lipid levels, regulate specific bacteria [such as those from genus Akkermansia, the family helicobacteraceae, and the Firmicutes-to-Bacteroidetes (F/B) ratio], optimize the composition of intestinal microbes (Wei et al., 2020; Li S. et al., 2021; Zhao et al., 2021), and then adjust lipid metabolism disorders induced by consumption of a high-fat diet (HFD). However, how SEJZ regulates dyslipidemia has not been investigated thoroughly. Moreover, the bioactive components and potential targets involved in the effects of SEJZ against hyperlipidemia are not known. Furthermore, examining GM function upon SEJZ treatment when investigating the influence of TCM formulations on intestinal disease is important (De Filippis et al., 2016).

Network pharmacology is a method for explaining complicated interactions between biological systems, medications, and diseases. It can be employed to determine synergistic effects in disease therapy by analyzing huge datasets and explaining the likely complicated bioactivity processes (Yang and Lao, 2019). We investigated the efficacious active ingredients and targets of SEJZ that could be predicted from network pharmacology. We also analyzed its key targets and signaling pathways for hyperlipidemia treatment. Then, the effects of SEJZ on the GM during hyperlipidemia were detected by 16S rRNA gene sequencing. We also determined the functional enrichment and signaling pathway enrichment of key genes using the Gene Ontology (GO) and Kyoto Encyclopedia of Genes and Genomes (KEGG) databases, respectively.

## Methods and Materials

### Collection of SEJZ and Hyperlipidemia Targets

We used “*Lonicera japonica* Thunb.” (Caprifoliaceae; *Lonicera japonica* branch, stem) as the keywords to harvest the targets of the active ingredients of SEJZ through the Traditional Chinese Medicine Systems Pharmacology Database and Analysis Platform (TCMSP) (https://old.tcmsp-e.com/tcmsp.php). The other compounds in the botanical drugs in SEJZ were obtained from the literature (Li et al., 2013; Bai et al., 2016; Lee et al., 2016; Nishida et al., 2016; Zhang et al., 2016; Yang et al., 2017a; Ohiri and Bassey, 2017; Wang et al., 2017; Wu et al., 2019b; Pan et al., 2019; Shi et al., 2019; Li et al., 2020a; Zhu et al., 2021). This information was entered into TCMSP to search for targets that corresponded to the active ingredients of SEJZ. The human genes associated with hyperlipemia were screened from GeneCards (www.genecards.org/), DrugBank (https://go.drugbank.com), and Online Mendelian Inheritance in Man (www.omim.org/) databases using “hyperlipemia” as the keyword to search for disease targets. Then, all the target proteins obtained above were converted into standardized gene names through the UniProt database (www.uniprot.org/).

### Construction of a Protein–Protein Interaction (PPI) Network

The therapeutic targets of SEJZ against hyperlipidemia were determined by mapping the targets of active compounds to hyperlipidemia-related targets. The therapeutic targets of SEJZ were entered into the Search Tool for the Retrieval of Interacting Genes/Proteins (STRING) database (https://string-db.org) and analyzed with organism species limited to *Homo sapiens* and a high confidence score (>0.9). The obtained PPI data were entered into Cytoscape 3.8.2 (https://cytoscape.org/) for visual analyses, and the topological parameter “Degree” was set to be two times greater than the median value (Zheng et al., 2020).

### Analysis of Signaling Pathway Enrichment Using the KEGG Database

We wished to clarify the potential signaling pathways of SEJZ in the treatment of hyperlipidemia. We used the Metascape database (https://metascape.org/gp/index.html#/main/step1) to conduct analyses of signaling pathway enrichment *via* the KEGG database. Species were limited to *Homo sapiens* and the diagram for signaling pathway enrichment was obtained using R 3.5.2. (R Institute for Statistical Computing, Vienna, Austria).

### Chemicals and Materials

*E. senticosus* (*Rupr.* and *Maxim*.), *Maxim* (Araliaceae; *E. senticosus* radix and rhizome) (lot number Z20180312), *C. pinnatifida* Bunge. (Rosaceae; Crataegus pinnatifida fruit) (Z20180306), and *L. japonica* Thunb. (Caprifoliaceae; *Lonicera japonica* branch, stem) (lot# Z20180215) were purchased from Harbin Pharmaceutical Group Shiyitang (Harbin, China). *A. auricula* was obtained from the Mudanjiang planting area and identified by Professor Wang Zhenyue of Heilongjiang University of Traditional Chinese Medicine (HUTCM; Harbin, China). Standard chow and a HFD [63.6% standard chow + 1.2% cholesterol + 20% sucrose + 15% lard + 0.2% sodium cholate; certificate number: SCXK (Beijing) 2014-0010] were provided by the laboratory animal center of HUTCM.

### Preparation of SEJZ Extract

*E. senticosus* (*Rupr.* and *Maxim.*), *Maxim* (Araliaceae; *E. senticosus* radix and rhizome), *L. japonica* Thunb. (Caprifoliaceae; *Lonicera japonica* branch, stem), *C. pinnatifida* Bunge. (Rosaceae; Crataegus pinnatifida fruit), and *A. auricula* were prepared at a ratio of 6:4:4:3 (*w/w/w/w*): 265 g of *E. senticosus* (*Rupr.* and *Maxim.*), *Maxim.* (Araliaceae; *E. senticosus* radix and rhizome.), 177 g of *L. japonica* Thunb. (Caprifoliaceae; *Lonicera japonica* branch, stem), 177 g of *C. pinnatifida* Bunge. (Rosaceae; Crataegus pinnatifida fruit), and 133 g of *A. auricula*. After mixing, all botanical drugs were soaked in 12 volumes of water for 30 min and heated at temperature 100°C to prepare the decoction. The formulation was decocted twice for 3 h, and the two decoctions were condensed and refluxed after combination; the compound extract was concentrated at 55°C and 30 r/min by rotary evaporator, and a 236.1 g of compound extract was prepared by freeze drying. First, the experimental drug solution was prepared according to the concentration required for high-dose administration. Using the weight of 24 Sprague–Dawley (SD) rats in the administration group, we determined that 25 g of the compound extract of the crude drug was required. Subsequently, 40 g of the extract was taken to account for potential experiment error. To meet the high dose (7.32 g/mg), we required 13.3 g of the compound extract. The volume of intragastric administration of the high-dose compound extract was 1.25 ml for each mouse, making the concentration of high dose 1.33g/ml. Therefore, the 40 g of compound extract was resuspended in 30.2 ml of water. To reduce experimental error, the high-dose solution was used as the mother solution. Subsequently, 10 and 5 ml of this high-dose mother solution were diluted with 20 and 19 ml of water, respectively, to prepare medium- and low-dose experimental solutions, respectively. Compound extract ratio and similar basic quality parameters, qualitative and quantitative assessments, prepared ratio and extraction process (Supplementary Material S3).

### Animal Experiments

The Experimental Animal Committee of HUTCM approved (20190921) the study protocol. Specific pathogen–free male SD rats (180 ± 20 g) were purchased from the Experimental Animal Center of HUTCM [certificate number: SYXK (Heilongjiang) 2018-007]. Rats were maintained in a controlled environment at 22 ± 3°C and humidity of 53 ± 7%. Rats were exposed to natural lighting conditions and allowed to acclimatize to their environment for 3 days. All rats had free access to chow and water.

Forty male SD rats were divided randomly into two groups: a blank control (BC) group (*n* = 8) and model group (MG) (*n* = 32). Rats in the BC group were fed normal chow. The remainder of the rats was modeled for 2 weeks with a HFD similar to that employed for the development of hyperlipidemia in humans. Two weeks after modeling, blood was collected from the orbital vein after an overnight fast. If the serum level of TG, TC, and LDL in the MG was significantly higher than that in the BC group, then the model was deemed to have been created (Zhao et al., 2018). Thirty-two rats in the MG were modeled successfully, and the differences between the BC group and the MG were highly significant (*p* < 0.01) (Supplementary Material S2). Then, the rats were randomly divided into four subgroups with rats per group: MG, SEJZ high-dose (HD), SEJZ medium-dose (MD), and SEJZ low-dose (LD). Subsequently, the BC group continued to be fed a normal feed, and treatment groups were given a HFD and decoction of different doses. The SEJZ dose was converted according to the body surface area that the US Food and Drug Administration recommends to convert the dose from humans to animals. SEJZ was given (10 ml/group, i.g.) at 7 a.m. every day for 4 weeks. The model and BC groups were treated with the same amount of physiologic (0.9%) saline as the control group. Rats were weighed every week to determine the SEJZ dose and food intake.

### Sample Collection

All rats were given 3% pentobarbital sodium (45 mg/kg, i.p.) to avoid pain before killing. Blood samples were collected from the abdominal aorta and placed into disposable blood-collection tubes. Then, the blood samples were placed at room temperature for 30–60 min and centrifuged at 1788.8 × *g* for 10 min at 4°C. We took 0.3 g of large intestine (cecum) contents (Sheng et al., 2017; Wang C. et al., 2020), placed them in an aseptic centrifuge tube, and froze them rapidly in liquid nitrogen. Cecum contents were stored at −80°C for DNA extraction/analyses.

### Determination of Biochemical Parameters in Blood

An automated biochemical analyzer (Cobas C311; Roche, Basel, Switzerland) was used to measure the level of TC, TG, LDL, and HDL in the serum of rats in each group.

### 16S rRNA Gene Sequencing

Analyses of the 16S rRNA gene were undertaken to examine the therapeutic effects of SEJZ on the GM composition of rats. Total bacterial DNA was collected and amplified. Personalbio (Shanghai, China) sequenced the bacterial DNA on the MiSeq™ platform (Illumina, San Diego, CA, United States). Bioinformatics analysis was carried out on the basis of operational taxonomic units, which were clustered on the basis of sequence similarity of 100% according to Vsearch (https://github.com/torognes/Vsearch). QIIME 2019.4 (http://qiime.org/) was used to calculate the Chao1 Index, Observed Species Index, Shannon Index, Simpson’s Diversity Index, and Good’s Coverage Index to ascertain α-diversity. The unweighted UniFrac method was employed to carry out principal coordinate analysis (PCoA) and non-metric multidimensional scaling (NMDS) for β-diversity analysis. All processes were undertaken on GenesCloud (www.genescloud.cn/). PICRUSt2 (http://picrust.githubio/) was used to predict functionally enriched signaling pathways with regard to gut metabolism (Douglas et al., 2020).

## Results

### Information on Screened Targets and PPI Networks Relating to SEJZ and Hyperlipidemia

**FIGURE 1:**
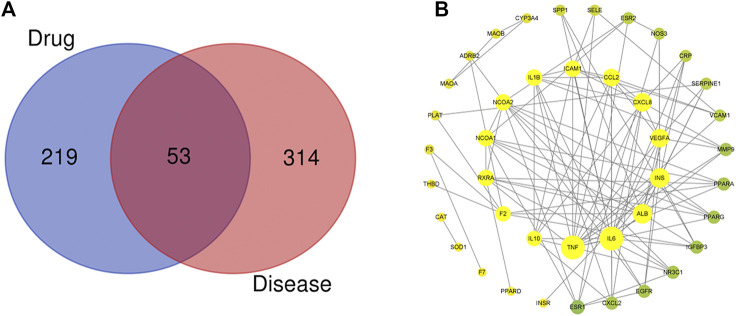
Characteristics of the targets of SEJZ in hyperlipidemia treatment were identified using network pharmacology. **(A)** Venn diagram of the common targets of SEJZ in hyperlipidemia treatment. **(B)** The protein–protein interaction (PPI) network is based on the targets of SEJZ. Nodes represent different proteins. Edges represent the association between proteins. The node size represents the strength of the association.

After removing duplicate targets, the databases yielded 272 pharmacological targets related to SEJZ. A total of 367 hyperlipidemia-related targets were obtained from the databases, and hyperlipidemia-related targets were mapped to 53 active ingredient targets. We used Venny 2.1 (https://bioinfogp.cnb.csic.es/tools/venny/) to obtain the therapeutic targets of SEJZ against hyperlipidemia (Figure 1A). Visualization of the PPI networks of SEJZ was achieved using the STRING database. Significantly more interactions were seen in the PPI network with 41 nodes and 135 edges (Figure 1B). Furthermore, the top 10 core genes screened were those for interleukin (IL)–1β, IL-6, tumor necrosis factor (TNF), IL-10, C-X-C motif chemokine ligand (CXCL) 8, monoamine oxidase B (MAOB), nitric oxide synthase (NOS) 3, epidermal growth factor receptor (EGFR), CXCL2, and intercellular adhesion molecule (ICAM) 1. These genes may be key targets for SEJZ to have a role in lowering of lipid concentrations.

### Enrichment of Signaling Pathways Using Core Targets

**FIGURE 2:**
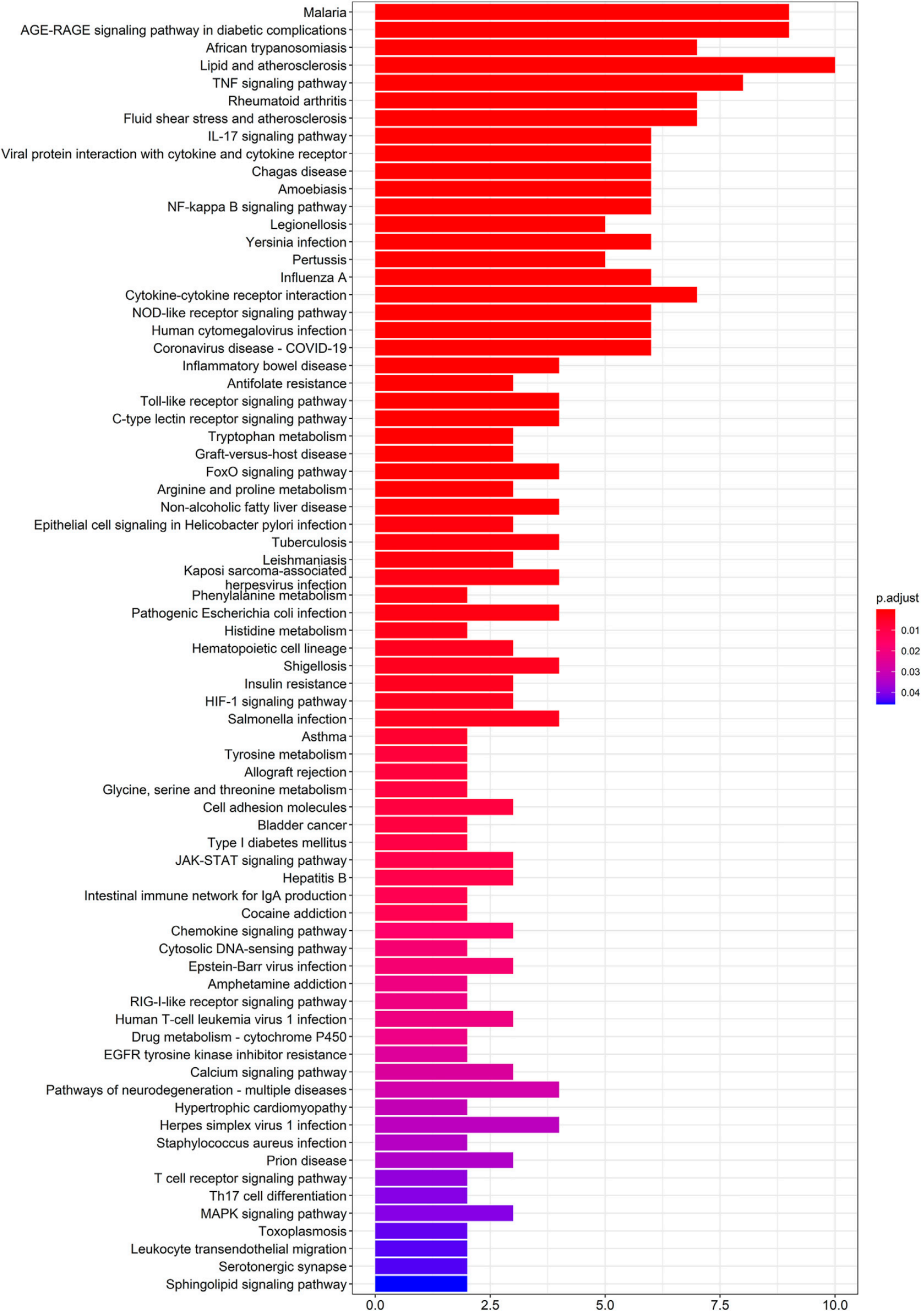
Analyses of pathway enrichment using the KEGG database.

To further elucidate the molecular mechanism of action of SEJZ on hyperlipidemia, analyses of pathway enrichment were undertaken on the 53 genes mentioned above using the KEGG database. Seventy-four metabolic pathways were identified (Figure 2).

### Effects of SEJZ on the Physiological and Biochemical Indices of Hyperlipidemia

**FIGURE 3:**
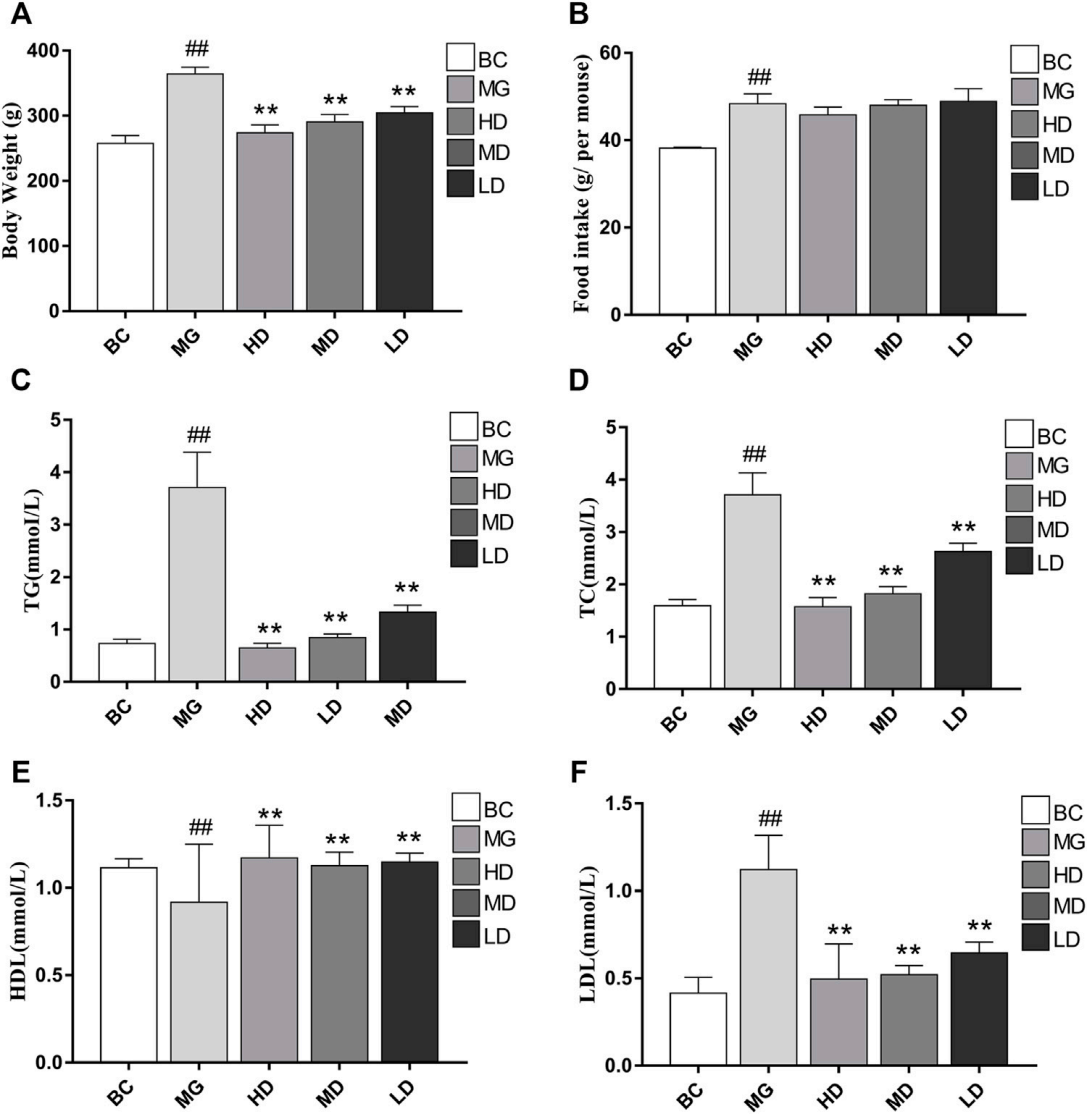
Effects of taking SEJZ for 4 weeks on the physiological and biochemical indices of model group -induced hyperlipidemia. **(A)** Body weight, **(B)** food intake, **(C)** TG, **(D)** TC, **(E)** HDL, and **(F)** LDL (*n* = 8, **p < 0.01 vs. MG; ^# #^ *p* < 0.01 vs. BC).

Visual signs and biochemical indicators were used to evaluate the therapeutic effect of SEJZ. At the beginning of the study, there were no significant differences in body weight or lipid indices. Two weeks after feeding MG rats with a HFD, the body weight and serum levels of TC, TG, and LDL in the BC group were significantly lower than those in the other groups. Simultaneously, the HDL level was significantly greater than that in the other groups (*p* < 0.01), which indicated that the model had been established (Supplementary Material S2). Upon SEJZ administration for 4 weeks, the differences in body weight and lipid indices between the BC group and MG were more significant (*p* < 0.01). Compared with the MG, the body weight and level of TC, TG, LDL, and HDL in serum were altered significantly in the treatment groups, especially in the HD group (*p* < 0.01), indicating that SEJZ could improve the hyperlipidemia caused by consumption of a HFD in a dose-dependent manner. The HD group had the best effect for lowering the lipid level in the blood (Figure 3).

### Modulatory Effects of SEJZ Upon Hyperlipidemia and the Role of the GM

**FIGURE 4:**
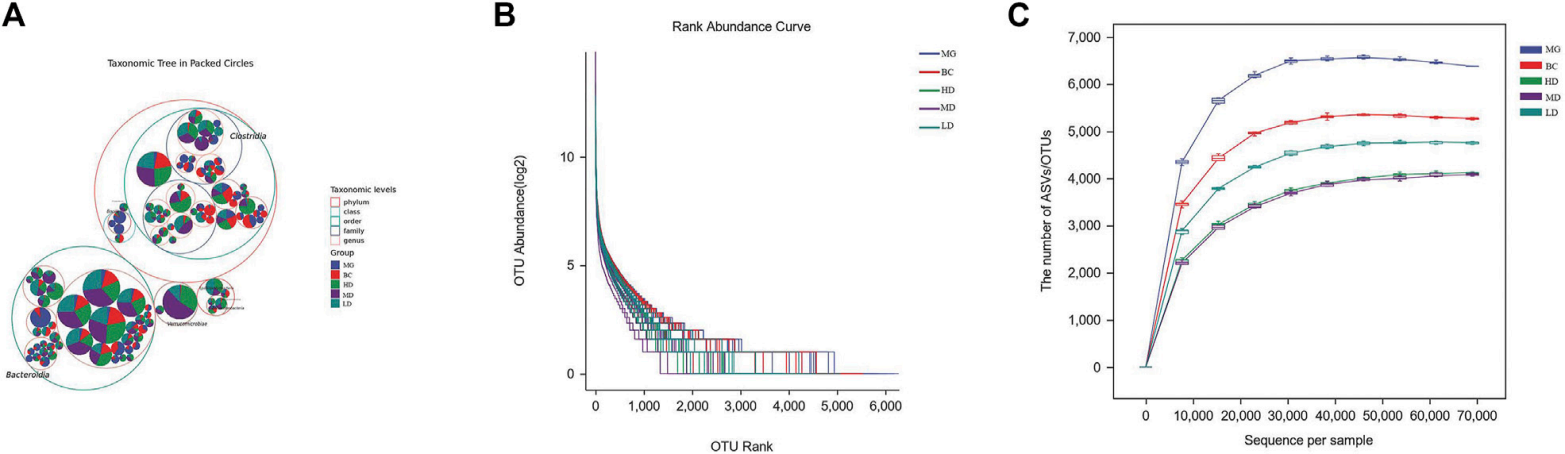
Effect of SEJZ treatment on the intestinal microflora of rats suffering from hyperlipidemia induced by consumption of a HFD. **(A)** Species composition. **(B)** Rarefaction curves. **(C)** Curve showing rank abundance.

An increasing number of studies have revealed that homeostasis of intestinal flora has a pivotal role in maintaining the integrity of the intestinal epithelial barrier and improving immune protection of the intestinal mucosa (Granado-Serrano et al., 2019). After completing quality-filtering steps, de-noising, and chimera removal, 3,909,195 high-quality sequences were obtained from 40 samples at a sequence similarity threshold of 100% for further analyses. Figure 4A shows the amplicon sequence variants (ASVs) of the top 100 most abundant genes. Most gut microbes could be annotated to the genus level. The slopes of the rarefaction curves were near saturation, which indicated that the sequencing depth was reasonable (Figure 4B). The curve showing rank abundance declined smoothly and became flat eventually (Figure 4C). The span of the horizontal coordinates was wide, which indicated that the species sample was rich and uniform.

**FIGURE 5:**
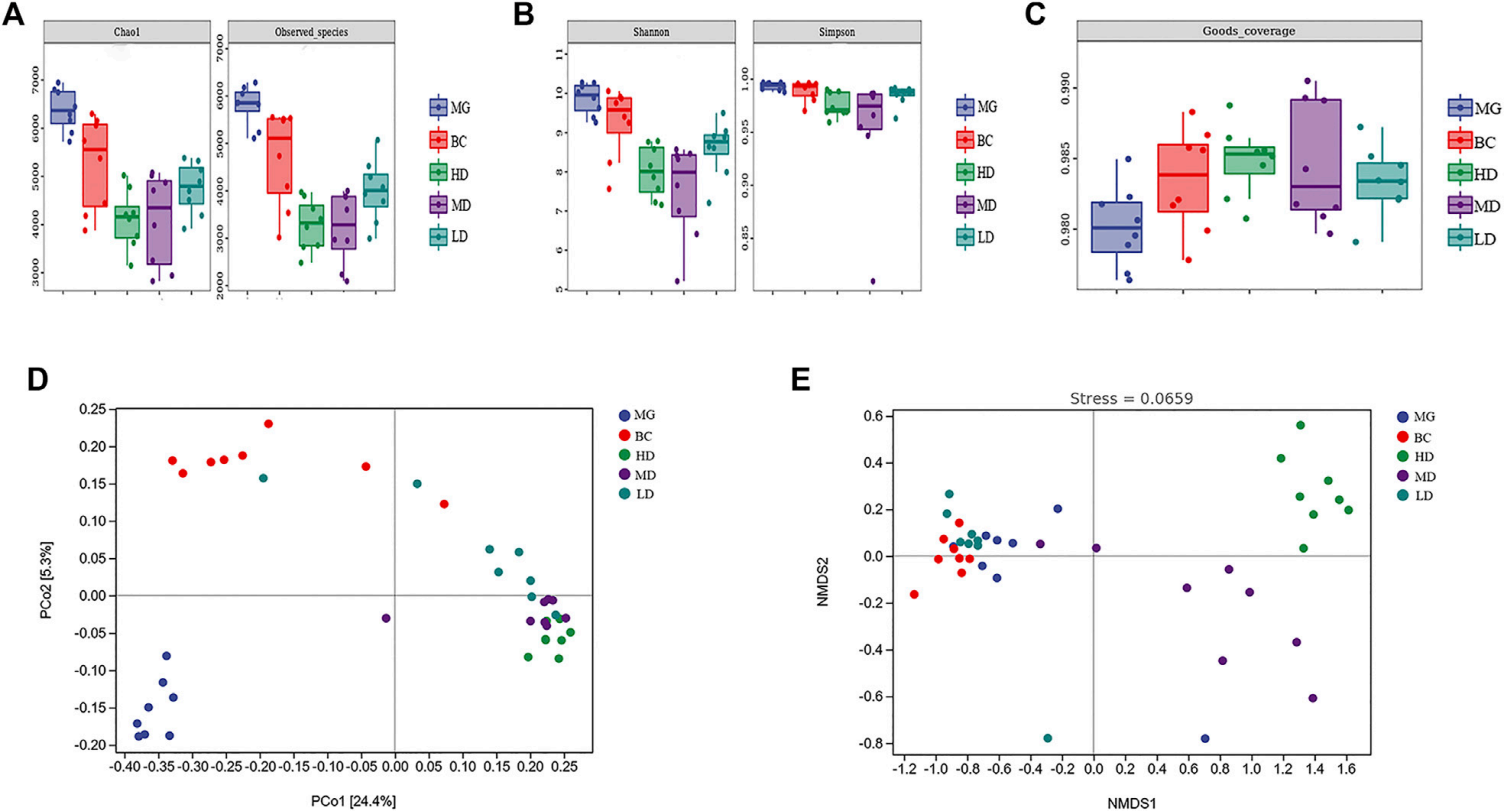
Species diversity. **(A)** Chao1 Index and Observed Species Index. **(B)** Shannon Index and Simpson Diversity Index **(C)** Good’s Coverage Index. PCoA **(D)** and NMDS **(E)** were applied on the basis of the unweighted UniFraq distance.

The α-diversity analysis showed that, compared with the BC group, the MG showed increases in the Chao1 Index, Observed Species Index, Shannon Diversity Index, and Simpson’s Diversity Index (Figures 5A,B). Consumption of a HFD could improve the richness and diversity of intestinal flora. Compared with the MG, the four indexes in the treatment group were decreased significantly, which indicated that SEJZ could inhibit the richness and diversity of the GM. This effect might be related to the bactericidal and anti-inflammatory effects of *E. senticosus* (*Rupr.* and *Maxim.*), *Maxim.* (Araliaceae; *E. senticosus* radix and rhizome.), and *L. japonica* Thunb (Caprifoliaceae; *Lonicera japonica* branch, stem) in SEJZ (Niu et al., 2015; Yang et al., 2021). The Good’s Coverage Index of each group was >98%, revealing that the sample coverage was high and that the sequencing results were reliable (Figure 5C). PCoA and NMDS were conducted on the basis of the ASVs for β-diversity analysis (Figures 5D,E). The projection distance of samples in the vertical coordinates was distinctly away between the MG and BC group, which indicated that the GM structure would be affected by a HFD. The GM structure of the treatment group shared the same tendency and was not closer to that of the BC group, which suggested that SEJZ did not significantly reverse the alterations in GM structure induced by consumption of a HFD. However, SEJZ did have a certain regulatory effect on GM structure. The stress value of NMDS results was small (<0.2), indicating that the NMDS results were reliable.

**FIGURE 6:**
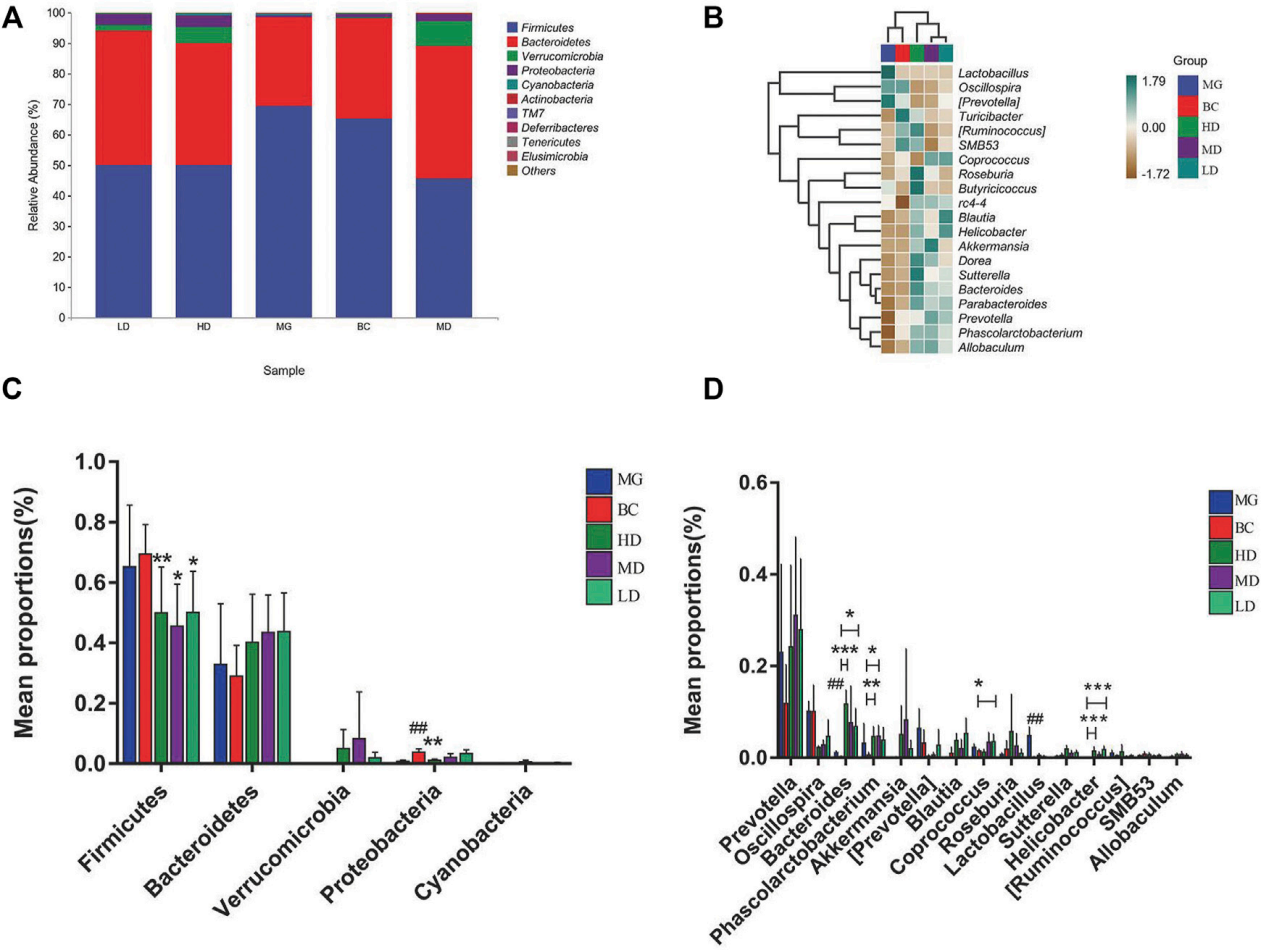
Bacteria with significant differences in abundance at different taxonomy levels. **(A)** Relative abundance of microbial species at the phylum level. **(B)** Heatmap of the relative abundance at the genus level. Differential abundance at the phylum level **(C)** and genus level **(D)**. One-way ANOVA, ^*^ *p <* 0.05 and ^**^ *p* < 0.01, *n* = 8.

The GM with significant differences between different groups was assessed by taxonomy. The top two phyla in the five groups were Firmicutes and Bacteroidetes (Figure 6A). In contrast to the MG, the F/B ratio in the treatment group was reduced significantly (Figure 6C). Figure 6B is a heatmap of the relative abundance of the top 20 genera among groups. *Lactobacillus* had no perceivable impact. At the genus level (Figure 6D), the abundance of bacteria associated with SCFAs, such as *Prevotella*, *Allobaculum*, *Lactobacillus*, *Roseburia* (Kasahara et al., 2018), *Phascolarctobacterium*, *Blautia*, and *Coprococcus*, was increased significantly, and the abundance of bacteria associated with intestinal-barrier repairs (Hu et al., 2017; Alexandre et al., 2018; Guo et al., 2021), such as *Akkermansia*, *Ruminococcus*, and *Oscillospira*, was reduced. *Verrucomicrobia* species and *Helicobacte*r species were found only in the treatment groups, which suggested that *Verrucomicrobia* species and *Helicobacter* species may be potential markers for SEJZ in hyperlipidemia treatment. Marked differences in GM composition between groups were observed.

**FIGURE 7:**
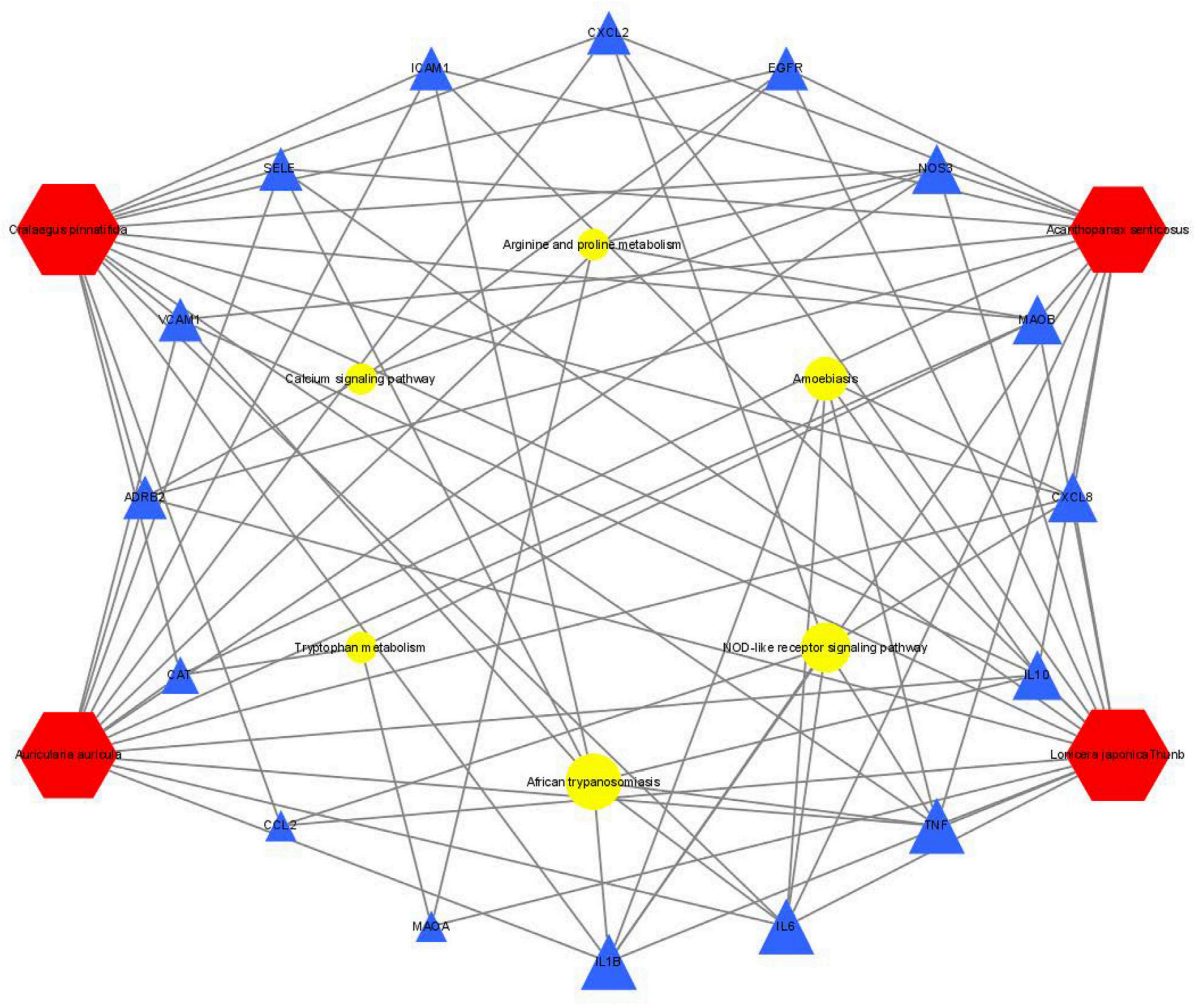
Integrated network plot for SEJZ in hyperlipidemia treatment.

We wished to verify the signaling pathways found through the previous network pharmacology of SEJZ upon hyperlipemia treatment. PICRUSt2 was used for functional analysis of the treatment group: 167 signaling pathways were found to be enriched. Six similar signaling pathways were obtained by mapping with 76 signaling pathways obtained from network pharmacology: “African trypanosomiasis”, “amoebiasis”, “arginine and proline metabolism”, “calcium signaling pathway”, “NOD-like receptor signaling pathway”, and “tryptophan metabolism”. On the basis of these six signaling pathways and the core genes obtained from network pharmacology, an integrated network plot of SEJZ for hyperlipidemia was mapped. The correlation results of each pathway and core genes are shown in Figure 7. The node representing African trypanosomiasis had the highest degree of association with the core genes and could be the main pathway by which SEJZ has a role in lowering lipid levels in the blood.

## Discussion

Six physiological and biochemical indicators (body weight, liver weight, as well as levels of TC, TG, HDL, and LDL) were selected to evaluate the lipid-lowering effect of SEJZ. TC is used to measure the cholesterol contained in lipoproteins in blood. HDL molecules remove fat and cholesterol from the body and protect the cardiovascular system. LDL molecules move cholesterol around the blood and deposit it on artery walls (Hua et al., 2020). TG molecules are vital components of adipose tissue, and TG content usually affects the levels of HDL and LDL in the body. We showed that the treatment groups alleviated body weight abnormalities and lipid composition in serum (TC, TG, HDL, and LDL) to varying degrees, with the HD group (22.95 g/kg/day) having the most significant effect. High-dose SEJZ had a potent lipid-lowering effect.

Network pharmacology and 16S rRNA gene sequencing were used to investigate the molecular mechanism of SEJZ in hyperlipidemia treatment. Fifty-three active ingredients of SEJZ, when used in the treatment of hyperlipidemia, were screened. Among them, apigenin, kaempferol, and quercetin were the core active components. They belong to the flavonol class of flavonoids and are present in multiple TCM formulations. These flavonols can improve the metabolism of lipids and glucose and reduce the level of cholesterol and TG in serum (Chang et al., 2011; Sekhon-Loodu et al., 2015; Xu et al., 2021). Flavonols can also increase the GM abundance and increase the F/B ratio, and their effect on bacteria of the phylum *Bacteroides* is potent, which may be related to SCFA generation (Mbikay et al., 2018; Wang T. et al., 2020).

Seventy-eight signaling pathways were enriched according to analyses carried out using the KEGG database. Six of these signaling pathways were consistent with the metabolic pathways predicted using PICRUSt2. Among the six enriched signaling pathways, the “African trypanosomiasis signaling pathway” was the most enriched. It has been reported that a variable surface glycoprotein blocks the treatment of African trypanosomiasis. It can stimulate macrophages to release various proinflammatory factors (TNFα, IL-6, IL-1, ICAM1, vascular cell adhesion molecule-1). Overexpression of proinflammatory factors can activate overexpression of the suppressor of cytokine signaling-3, increase the production of mature sterol regulatory element-binding protein-1, aggravate TG deposition, and cause abnormal lipid metabolism (Tan et al., 2017). In addition, Rouzer and colleagues found that TG degradation in rabbits infected with *Trypanosoma brucei* was inhibited due to a lack of caspase activity in heparin-treated plasma (Rouzer and Cerami, 1980). Therefore, we speculate that this signaling pathway may be important in hyperlipidemia treatment using SEJZ.

We found that “Arginine and proline metabolism”, “calcium signaling pathway”, and “tryptophan metabolism” had little correlation with core genes, but research has demonstrated that amino acid metabolism can lead to metabolic diseases if it is abnormal (Wang et al., 2019). Tryptophan is an essential amino acid and involved in several important metabolic reactions. Tryptophan metabolites preserve vascular homeostasis by regulating critical cellular processes (proliferation, migration, differentiation, apoptosis, contractility, and senescence) and block lipid accumulation within arterial walls (Durante, 2020). GM changes contribute to the development of metabolic disorders (Derrien and van Hylckama Vlieg, 2015), including problems related to cholesterol metabolism. *Lactobacillus* species can be used as probiotics that can produce aryl hydrocarbon receptor ligands to accelerate tryptophan metabolism, thereby affecting SCFA production and reducing the cholesterol level. Abnormal metabolism of arginine and proline is also common in dyslipidemia (Shearer et al., 2008). Increased levels of arginine and proline are strongly linked to an increased risk of hypertriglyceridemia (Mook-Kanamori et al., 2014). Shao and colleagues (Shao et al., 2020) hypothesized that gut flora alleviates increased metabolism of arginine and proline by modulating bacteria of the genera *Bacteroides*, *Phascolarctobacterium*, *Prevotella*, *Roseburia*, and other bacteria. Excessive release of free fatty acids occurs if the TG level in the body is increased (Bush et al., 2012), and free fatty acids can significantly affect transduction of calcium signals, which might contribute to vascular dysfunction (Esenabhalu et al., 2003). As mentioned above, when the “African Trypanosomiasis Signaling Pathway” is blocked by the variable surface glycoprotein, macrophages will be stimulated to release more inflammatory factors, which will lead to TG deposition. Therefore, we speculated that the “African Trypanosomiasis Signaling Pathway” is the upstream signaling pathway of the calcium signaling Pathway. Moreover, the intervention of the “African Trypanosomiasis signaling Pathway” and then blockage of the calcium signaling pathway may also be an effective way to treat hyperlipidemia.

We evaluated, for the first time, the effect of SEJZ on hyperlipidemia induced by consumption of a HFD on the basis of 16S rRNA gene sequencing. The predominant bacteria in the GM of the treatment group were from the phyla Firmicutes and Bacteroidetes, and the F/B ratio was increased markedly in the treatment group compared with that in the MG which contributed to reducing excessive energy intake and fat deposition (Li YQ. et al., 2020). Furthermore, bacteria from Firmicutes can assist cellulose digestion and supplement nutrition and energy in the body. Some Gram-positive bacteria in Firmicutes can improve the body’s ability to resist pathogens and maintain the GM balance (Garneau et al., 2008). SEJZ treatment also significantly reduced the abundance of bacteria from the phylum Proteobacteria, which can cause gastritis and gastric ulcers (Shin et al., 2015; Kang et al., 2019). The HD group had an increased abundance of bacteria from the phylum Cyanobacteria, which contains a toxin that influences health (Carmichael, 1992; Wu et al., 2018) (Dixit and Suseela, 2013). Bacteria of the genus *Akkermansia* are symbiotic and found in healthy human intestines (Collado et al., 2007). They can maintain intestinal metabolic balance, restore the intestinal barrier and intestinal mucosal homeostasis (Derrien et al., 2017; Liu et al., 2017), and play a part in lipid lowering by lowering serum levels of proinflammatory cytokines (e.g., TNF-α) and lipopolysaccharide (Deng et al., 2020).

The abundance of bacteria from *Akkermansia* is often used as an index to evaluate physiological health in humans (Zhang et al., 2019). We showed that the abundance of bacteria of *Akkermansia* was increased significantly in the treatment group. SEJZ treatment also significantly increased the abundance of SCFA-producing bacteria, such as those of the genera *Roseburia* (Kasahara et al., 2018), *Phascolarctobacterium*, *Allobaculum*, *Prevotella* (Kong et al., 2019), and *Blautia*. SCFAs (especially butyric acid) provide energy to colonic epithelial cells and regulate gene expression (Ritzhaupt et al., 1998), inhibit activation of nuclear factor-kappa B, and have an anti-inflammatory role (Segain et al., 2000) (Zhang et al., 2015). SEJZ treatment reduced the change in GM structure by adjusting the abundance of *Lactobacillus* species. Studies have shown that *Lactobacillus* species can affect cholesterol oxidase and fatty acid synthase, as well as redistribute TG and cholesterol in the liver and blood, thereby improving lipid metabolism disorders and intestinal hepatic circulation of bile acid (Wu et al., 2019a; Guandalini and Sansotta, 2019). In addition, SEJZ treatment reduces the abundance of *Oscillospira* species, which degrade glycan components in human intestines (Konikoff and Gophna, 2016). *Helicobacter* species colonize the human gastric mucosa, can cause gastric cancer and peptic ulcers, and can alter the lipid profile in the body (Yang et al., 2014; Obaidat and Roess, 2019). We showed that, compared with the MG, the abundance of *Helicobacter* species was increased significantly in the SEJZ treatment group. Fortunately, the prevalence of *Helicobacter* species is declining worldwide (Burucoa and Axon, 2017).

## Conclusion

SEJZ could relieve the hyperlipidemia caused by consumption of a HFD through upregulation of metabolic pathways related to restoration of the intestinal barrier. Several signaling pathways affected by SEJZ in the treatment of hyperlipidemia were identified by network pharmacology and 16S rRNA gene sequencing. Our data provide new insights on the lipid-lowering effect of SEJZ.

## Data Availability

The authors acknowledge that the data presented in this study must be deposited and made publicly available in an acceptable repository, prior to publication. Frontiers cannot accept an article that does not adhere to our open data policies.

## Abbreviations

SEJZ, Shenerjiangzhi formulation; HFD, high-fat diet; MG, model group; HD, high dose; MD, medium dose; LD, low dose; KEGG, Kyoto Encyclopedia of Genes and Genomes; GO, Gene Ontology; SCFAs, short-chain fatty acids; PPI, protein–protein interaction; TG, triglycerides; TC, total cholesterol; LDL, low-density lipoprotein; HDL, high-density lipoprotein; TCM, traditional Chinese medicine; TCMSP, Traditional Chinese Medicine Systems Pharmacology database and analysis platform; OMIM, online mendelian inheritance in man; STRING, Search Tool for the Retrieval of Interacting Genes/Proteins; PCoA, principal coordinate analysis; NMDS, non-metric multidimensional scaling; IL, interleukin; TNF, tumor necrosis factor; CXCL, C-X-C motif chemokine ligand; MAOB, monoamine oxidase B; NOS, nitric oxide synthase; EGFR, epidermal growth factor receptor; ICAM, intercellular adhesion molecule; i.p., intraperitoneal injection; GM, gut microbiota.
