# Supplementary material for: Mechanism of Action of Shenerjiangzhi Formulation on Hyperlipidemia Induced by Consumption of a High-Fat Diet in Rats Using Network Pharmacology and Analyses of the Gut Microbiota
Source: Front Pharmacol. 2022 Apr 5;13:745074. doi: 10.3389/fphar.2022.745074 (PMC9016632; doi:10.3389/fphar.2022.745074)
Supplement: Supplementary file 1 [file DataSheet3.zip › S3/CWS_Editorial_Certificate.pdf]

Charlesworth Author Services

EDITORIAL CERTIFICATE

This document certifies that the manuscript below was edited for correct English language usage, grammar, punctuation and spelling by qualified native English speaking editors at Charlesworth Author Services.

Paper Title:

Based on Network pharmacology and Gut Microbiota Analysis to Explore the mechanism of SHENERJIANGZHI Formula on Hyperlipidemia Induced by High Fat Diet in Rats

Author:

shuang zhang

Date certificate issued:

January 24, 2022

cwauthors.com
