# Supplementary material for: Mechanism of Action of Shenerjiangzhi Formulation on Hyperlipidemia Induced by Consumption of a High-Fat Diet in Rats Using Network Pharmacology and Analyses of the Gut Microbiota
Source: Front Pharmacol. 2022 Apr 5;13:745074. doi: 10.3389/fphar.2022.745074 (PMC9016632; doi:10.3389/fphar.2022.745074)
Supplement: Supplementary file 1 [file DataSheet3.zip › S3/S3 1.7.docx]

**Prepared Ratio**

**DESIGN-EXPERT**

**A**: *Crataegus pinnatifida* Bunge; **B:** *Auricularia auricula***; C:** *Acanthopanax senticosus;****D****: Lonicera japonica* Thunb;

**Table 1 The code of Design-Expert**

| Factor | code | | | | |
| --- | --- | --- | --- | --- | --- |
|  | -2 | -1 | 0 | 1 | 2 |
| A | 9 | 9.75 | 10.5 | 11.25 | 12 |
| B | 3 | 4.75 | 6.5 | 8.25 | 10 |
| C | 9 | 13.5 | 18 | 22.5 | 27 |
| D | 6 | 8.25 | 10.5 | 12.75 | 15 |

**Table 2 The expertment result of Design-Expert**

| sequence | A | B | C | D | CHOL(Y1) | TG(Y2) | HDL(Y3) | LDL(Y4) |
| --- | --- | --- | --- | --- | --- | --- | --- | --- |
| 1 | 9.75 | 4.75 | 13.5 | 12.75 | 1.73 | 0.73 | 1.2 | 0.6 |
| 2 | 9.75 | 8.25 | 13.5 | 12.75 | 1.64 | 0.77 | 1.17 | 0.55 |
| 3 | 10.5 | 6.5 | 27 | 10.5 | 1.17 | 0.56 | 1 | 0.39 |
| 4 | 11.25 | 4.75 | 13.5 | 12.75 | 1.43 | 0.57 | 0.96 | 0.45 |
| 5 | 9.75 | 8.25 | 22.5 | 8.25 | 1.58 | 0.69 | 1.25 | 0.58 |
| 6 | 10.5 | 6.5 | 18 | 10.5 | 1.67 | 0.73 | 1.61 | 0.51 |
| 7 | 11.25 | 8.25 | 13.5 | 8.25 | 1.56 | 0.77 | 1.23 | 0.51 |
| 8 | 10.5 | 6.5 | 18 | 6 | 1.82 | 0.9 | 1.41 | 0.56 |
| 9 | 11.25 | 8.25 | 13.5 | 12.75 | 1.6 | 0.55 | 1.39 | 0.38 |
| 10 | 11.25 | 8.25 | 22.5 | 8.25 | 1.67 | 0.56 | 1.2 | 0.49 |
| 11 | 10.5 | 6.5 | 18 | 10.5 | 1.63 | 0.77 | 1.18 | 0.45 |
| 12 | 11.25 | 4.75 | 22.5 | 12.75 | 1.45 | 0.73 | 1.37 | 0.33 |
| 13 | 10.5 | 10 | 18 | 10.5 | 1.5 | 0.62 | 1.14 | 0.46 |
| 14 | 10.5 | 6.5 | 18 | 10.5 | 1.7 | 0.75 | 1.18 | 0.47 |
| 15 | 12 | 6.5 | 18 | 10.5 | 1.4 | 0.5 | 1.22 | 0.34 |
| 16 | 10.5 | 6.5 | 18 | 10.5 | 1.61 | 0.78 | 1.17 | 0.45 |
| 17 | 10.5 | 6.5 | 9 | 10.5 | 1.32 | 0.7 | 1.19 | 0.63 |
| 18 | 10.5 | 6.5 | 18 | 10.5 | 1.64 | 0.67 | 1.22 | 0.47 |
| 19 | 11.25 | 8.25 | 22.5 | 12.75 | 1.42 | 0.51 | 1.36 | 0.25 |
| 20 | 9.75 | 4.75 | 22.5 | 12.75 | 1.49 | 0.69 | 1.36 | 0.5 |
| 21 | 10.5 | 6.5 | 18 | 10.5 | 1.6 | 0.8 | 1.19 | 0.46 |
| 22 | 11.25 | 4.75 | 22.5 | 8.25 | 1.35 | 0.64 | 1.21 | 0.42 |
| 23 | 9.75 | 4.75 | 22.5 | 8.25 | 1.67 | 0.88 | 1.3 | 0.6 |
| 24 | 10.5 | 3 | 18 | 10.5 | 1.74 | 0.79 | 1.41 | 0.55 |
| 25 | 9.75 | 4.75 | 13.5 | 8.25 | 1.93 | 1.08 | 1.29 | 0.68 |
| 26 | 11.25 | 4.75 | 13.5 | 8.25 | 1.71 | 0.79 | 1.23 | 0.54 |
| 27 | 9.75 | 8.25 | 22.5 | 12.75 | 1.45 | 0.58 | 1.24 | 0.44 |
| 28 | 9 | 6.5 | 18 | 10.5 | 1.89 | 0.9 | 1.65 | 0.7 |
| 29 | 9.75 | 8.25 | 13.5 | 8.25 | 1.78 | 0.86 | 1.31 | 0.65 |
| 30 | 10.5 | 6.5 | 18 | 15 | 1.45 | 0.764 | 1.29 | 0.34 |

Table 3 Anova table of effect surface quadratic model Y1

|  | | | Sum of | |  | | | Mean | | | F | | | p-value | |  | | |
| --- | --- | --- | --- | --- | --- | --- | --- | --- | --- | --- | --- | --- | --- | --- | --- | --- | --- | --- |
| Source | | | Squares | | df | | | Square | | | Value | | | Prob > F | |  | | |
| Model | | | 0.74 | | 14 | | | 0.053 | | | 6.09 | | | < 0.0001 | | significant | | |
| A | | | 0.18 | | 1 | | | 0.18 | | | 20.98 | | | < 0.0001 | |  | | |
| B | | | 0.012 | | 1 | | | 0.012 | | | 1.34 | | | 0.2645 | |  | | |
| C | | | 0.11 | | 1 | | | 0.11 | | | 12.52 | | | 0.003 | |  | | |
| D | | | 0.13 | | 1 | | | 0.13 | | | 15.35 | | | 0.0014 | |  | | |
| AB | | | 0.029 | | 1 | | | 0.029 | | | 3.35 | | | 0.0873 | |  | | |
| AC | | | 0.014 | | 1 | | | 0.014 | | | 1.59 | | | 0.2266 | |  | | |
| AD | | | 3.79E-03 | | 1 | | | 3.79E-03 | | | 0.44 | | | 0.5179 | |  | | |
| BC | | | 8.57E-03 | | 1 | | | 8.57E-03 | | | 0.99 | | | 0.3353 | |  | | |
| BD | | | 2.61E-04 | | 1 | | | 2.61E-04 | | | 0.03 | | | 0.8643 | |  | | |
| CD | | | 8.51E-04 | | 1 | | | 8.51E-04 | | | 0.099 | | | 0.7579 | |  | | |
| A^2 | | | 2.93E-03 | | 1 | | | 2.93E-03 | | | 0.34 | | | 0.5689 | |  | | |
| B^2 | | | 3.51E-04 | | 1 | | | 3.51E-04 | | | 0.041 | | | 0.843 | |  | | |
| C^2 | | | 0.22 | | 1 | | | 0.22 | | | 25.46 | | | 0.0001 | |  | | |
| D^2 | | | 1.53E-03 | | 1 | | | 1.53E-03 | | | 0.18 | | | 0.6796 | |  | | |
| Residual | | | 0.13 | | 15 | | | 8.64E-03 | | |  | | |  | |  | | |
| Lack of fit | | | 0.12 | | 10 | | | 0.012 | | | 8.53 | | | 0.1044 | | not significant | | |
| Pure Error 7.18E-03 | | | | | 5 | | | 1.44E-03 | | |  | | |  | |  | | |
| Cor Total 0.87 | | | | | 29 | | |  | | |  | | |  | |  | | |
|  | | | | | Table 4 Anova table of effect surface quadratic model Y2 | | | | | | | | | | |  | | |
| Sum of | | | | | Mean F p-value | | | | | | | | | | |  | | |
| Source Squares | | | | | df Square Value Prob > F | | | | | | | | | | |  | | |
| Model | | 0.45 | 14 | | 0.032 | | | 9.47 | | < 0.0001 | | | | significant |  |  |  |  |
| A | | 0.16 | 1 | | 0.16 | | | 47.02 | | < 0.0001 | | | |  |  |  |  |  |
| B | | 0.052 | 1 | | 0.052 | | | 15.51 | | 0.0013 | | | |  |  |  |  |  |
| C | | 0.052 | 1 | | 0.052 | | | 15.46 | | 0.0013 | | | |  |  |  |  |  |
| D | | 0.082 | 1 | | 0.082 | | | 24.1 | | 0.0002 | | | |  |  |  |  |  |
| AB | | 1.15E-03 | 1 | | 1.15E-03 | | | 0.34 | | 0.5681 | | | |  |  |  |  |  |
| AC | | 9.36E-03 | 1 | | 9.36E-03 | | | 2.77 | | 0.117 | | | |  |  |  |  |  |
| AD | | 7.94E-03 | 1 | | 7.94E-03 | | | 2.35 | | 0.1464 | | | |  |  |  |  |  |
| BC | | 8.35E-03 | 1 | | 8.35E-03 | | | 2.47 | | 0.137 | | | |  |  |  |  |  |
| BD | | 2.52E-03 | 1 | | 2.52E-03 | | | 0.74 | | 0.402 | | | |  |  |  |  |  |
| CD | | 0.026 | 1 | | 0.026 | | | 7.58 | | 0.0148 | | | |  |  |  |  |  |
| A^2 | | | 5.02E-03 | | 1 | 5.02E-03 | | 1.48 | | | | 0.2422 | |  | | |  |  |
| B^2 | | | 4.18E-03 | | 1 | 4.18E-03 | | 1.24 | | | | 0.2837 | |  | | |  |  |
| C^2 | | | 0.024 | | 1 | 0.024 | | 7.18 | | | | 0.0171 | |  | | |  |  |
| D^2 | | | 0.011 | | 1 | 0.011 | | 3.3 | | | | 0.0895 | |  | | |  |  |
| Residual | | | 0.051 | | 15 | 3.38E-03 | |  | | | |  | |  | | |  |  |
| Lack of Fi t | | | 0.041 | | 10 | 4.07E-03 | | 2.02 | | | | 0.2271 | | not significant | | |  |  |
| Pure Error | | | 0.01 | | 5 | 2.02E-03 | |  | | | |  | |  | | |  |  |
| Cor Total | | | 0.5 | | 29 |  | |  | | | |  | |  | | |  |  |

Table 5 Anova table of effect surface quadratic model Y3

| Source | Sum of  Squares | df | Mean  Square | F  Value | p-value  Prob > F |  |
| --- | --- | --- | --- | --- | --- | --- |
|  |  |  |  |  |  |  |
| Model | 0.33 | 14 | 0.023 | 47.23 | < 0.0001 | significant |
| A | 0.16 | 1 | 0.16 | 317.96 | < 0.0001 |  |
| B | 9.15E-03 | 1 | 9.15E-03 | 18.45 | 0.0006 |  |
| C | 0.061 | 1 | 0.061 | 122.48 | < 0.0001 |  |
| D | 0.083 | 1 | 0.083 | 166.9 | < 0.0001 |  |
| AB | 3.23E-04 | 1 | 3.23E-04 | 0.65 | 0.4323 |  |
| AC | 4.24E-05 | 1 | 4.24E-05 | 0.086 | 0.7739 |  |
| AD | 9.21E-04 | 1 | 9.21E-04 | 1.86 | 0.1931 |  |
| BC | 6.06E-04 | 1 | 6.06E-04 | 1.22 | 0.2866 |  |
| BD | 3.89E-03 | 1 | 3.89E-03 | 7.85 | 0.0134 |  |
| CD | 1.80E-03 | 1 | 1.80E-03 | 3.62 | 0.0763 |  |
| A^2 | 4.96E-03 | 1 | 4.96E-03 | 10.01 | 0.0064 |  |
| B^2 | 2.36E-03 | 1 | 2.36E-03 | 4.76 | 0.0454 |  |
| C^2 | 3.15E-03 | 1 | 3.15E-03 | 6.35 | 0.0236 |  |
| D^2 | 4.67E-04 | 1 | 4.67E-04 | 0.94 | 0.3475 |  |
| Residual | 7.44E-03 | 15 | 4.96E-04 |  |  |  |
| Lack of Fi t | 5.12E-03 | 10 | 5.12E-04 | 1.1 | 0.4856 | not significant |
| Pure Error | 2.32E-03 | 5 | 4.64E-04 |  |  |  |
| Cor Total | 0.34 | 29 |  |  |  |  |


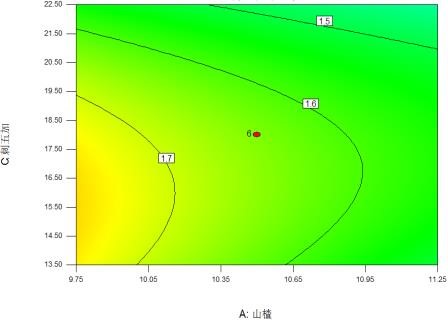

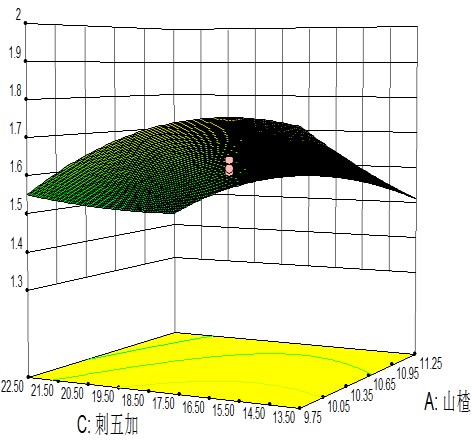


**Fig** 1 Effects of *Crataegus pinnatifida* Bunge and Acanthopanax senticosus on CHOL


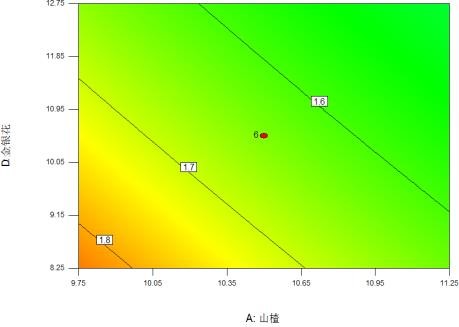

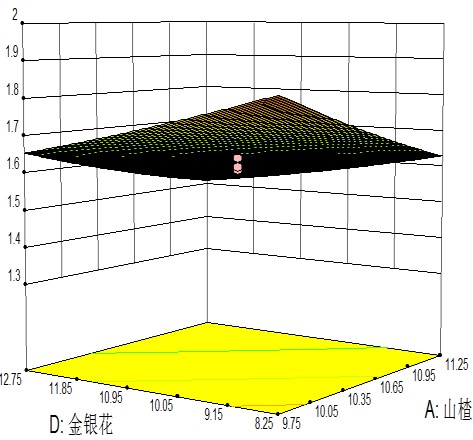


**Fig** 2 Effects of *Crataegus pinnatifida* Bungeand*Lonicera japonica* Thunb on CHOL


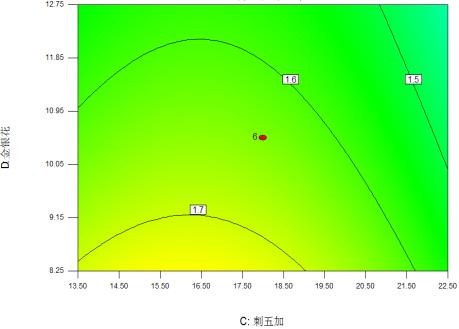

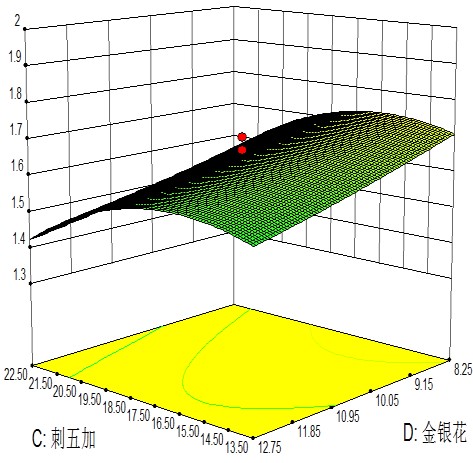


**Fig** 3 Effects of *Lonicera japonica* Thunb and *Acanthopanax senticosus* on CHOL


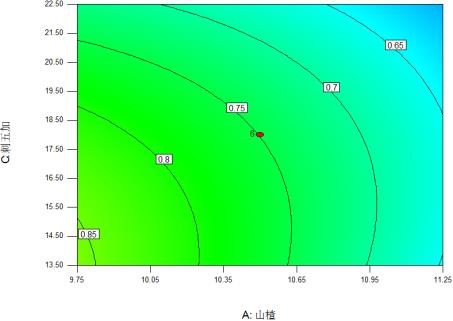

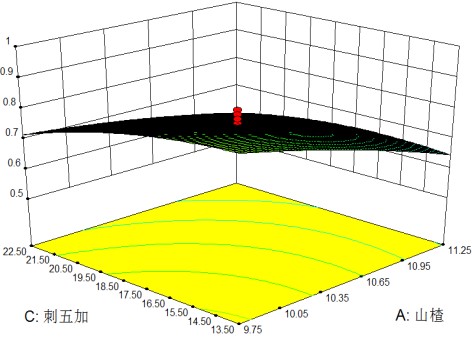


**Fig** 4 Effects of *Crataegus pinnatifida* Bunge and *Acanthopanax senticosus* on TG


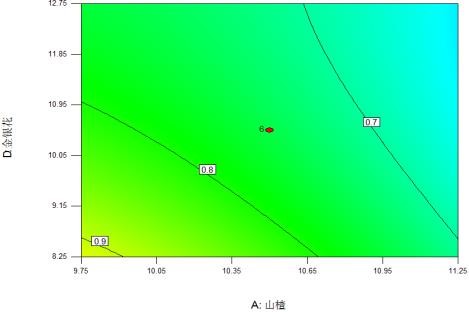

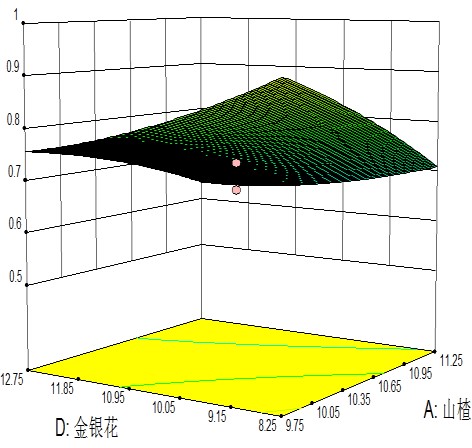


**Fig** 5 Effects of *Crataegus pinnatifida* Bunge and *Lonicera japonica* Thunb on TG


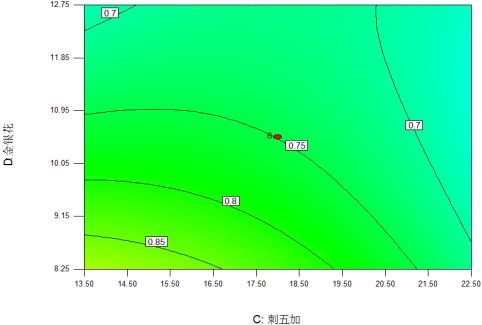

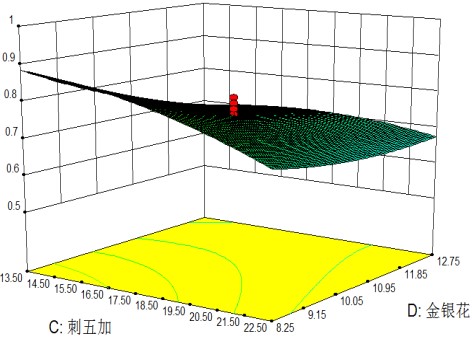


**Fig** 6 Effects of *Lonicera japonica* Thunb and *Acanthopanax senticosus* on TG


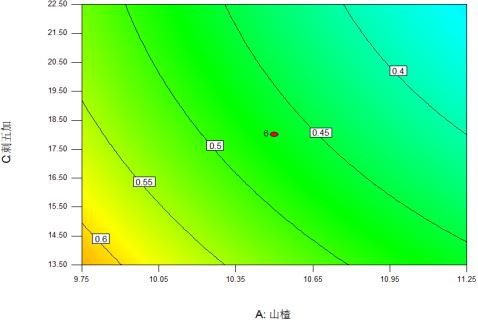

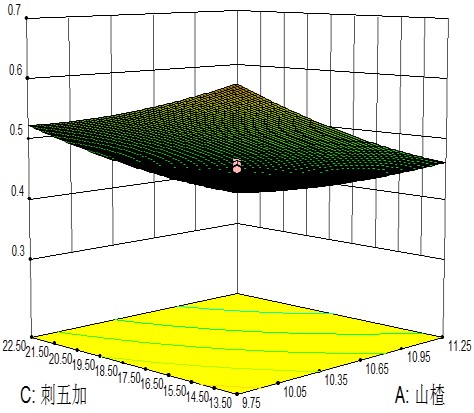


**Fig** 7 Effects of *Crataegus pinnatifida* Bunge and *Acanthopanax senticosus* on LDL


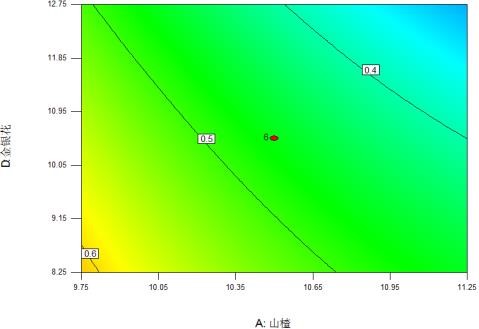

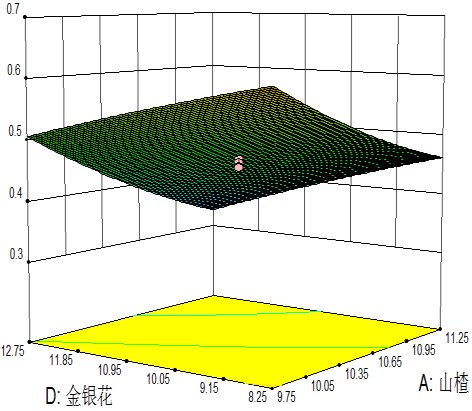


**Fig** 8 Effects of *Crataegus pinnatifida* Bunge and *Lonicera japonica* Thunb on LDL


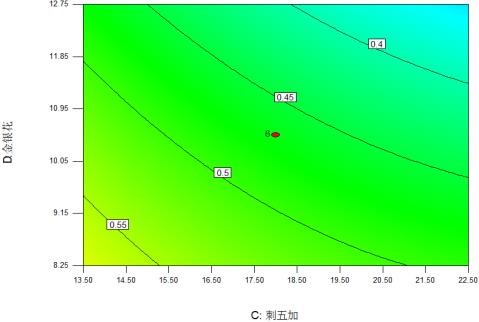

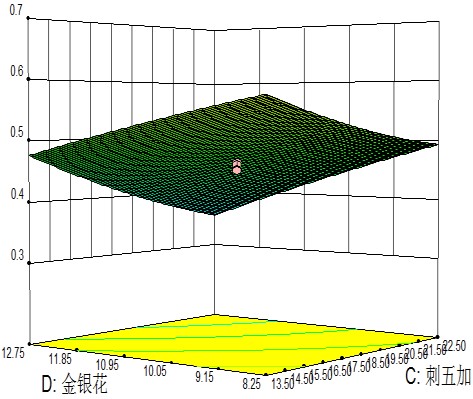


**Fig** 9 Effects of *Lonicera japonica* Thunb and *Acanthopanax senticosus* on LDL

**Extraction process**

**The orthogonal design**

Table 6 Table of orthogonal experimental factor

| group | The ratio of material to liquid | number of extractions | extraction time |
| --- | --- | --- | --- |
| 1 | 8 | 1 | 1 |
| 2 | 10 | 2 | 2 |
| 3 | 12 | 3 | 3 |

Table 7 Table of orthogonal experiment results

| Factor | The ratio of material to liquid | number of extractions | extraction time | Blank | result |
| --- | --- | --- | --- | --- | --- |
| experiment1 | 8 | 1 | 1 | 1 | 1.55 |
| experiment2 | 8 | 2 | 2 | 2 | 0.82 |
| experiment3 | 8 | 3 | 3 | 3 | 0.83 |
| experiment4 | 10 | 1 | 2 | 3 | 1.39 |
| experiment5 | 10 | 2 | 3 | 1 | 0.81 |
| experiment6 | 10 | 3 | 1 | 2 | 0.96 |
| experiment7 | 12 | 1 | 3 | 2 | 1.31 |
| experiment8 | 12 | 2 | 1 | 3 | 0.83 |
| experiment9 | 12 | 3 | 2 | 1 | 0.88 |
| mean value 1 | 1.067 | 1.417 | 1.113 | 1.080 |  |
| mean value 2 | 1.053 | 0.820 | 1.030 | 1.030 |  |
| mean value 3 | 1.007 | 0.890 | 0.983 | 1.017 |  |
| range | 0.060 | 0.597 | 0.130 | 0.063 |  |

Table 8 Analysis of variance

| Factor | Sum of square | df | F ratio | F Value | significant |
| --- | --- | --- | --- | --- | --- |
| The ratio of material to liquid | Squares | 2 | 0.857 | 19.000 |  |
| number of extraction**s** | 0.638 | 2 | 91.143 | 19.000 | * |
| extraction time | 0.026 | 2 | 3.714 | 19.000 |  |
| error | 0.010 | 2 |  |  |  |

Notice：* significant difference was indicated P＜0.05，F＞19。

**Qualitative and quantitative assessments**

**Thin layer chromatography (TLC)**


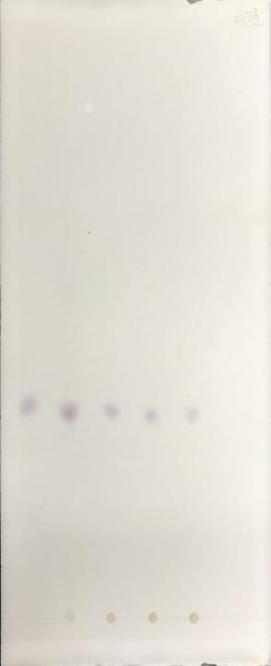

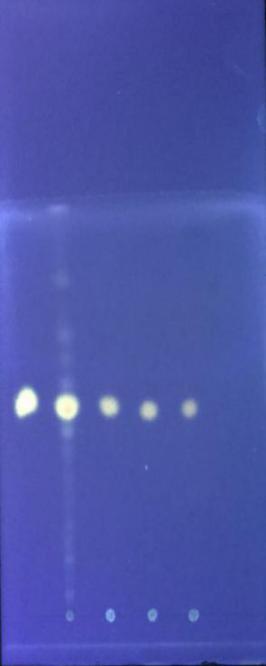


***Crataegus pinnatifida***

**A**


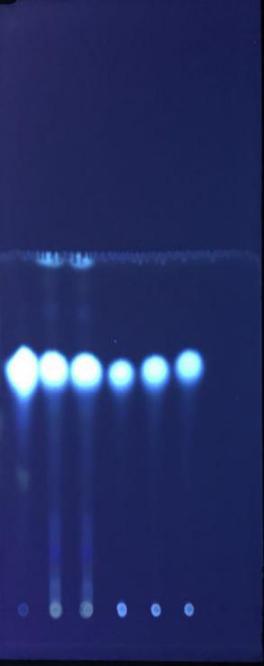

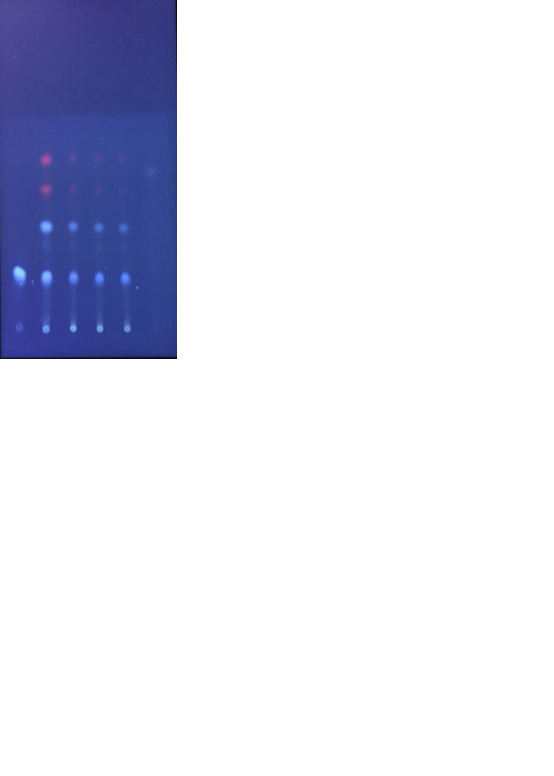


***Acanthopanax senticosus*** ***Lonicera japonica* Thunb**

C

B

**Fig.10: *Crataegus pinnatifida(A)* 、*Lonicera japonica* Thunb(C)** :The first point is standards; the second point is verb; the third to the fifth point is SEJZ formulation; . the sixth point is the negative control. ***Acanthopanax senticosus(B):*** The first point is standards; the second point is verb; the third point is standard verb; the third point to the sixth point is SEJZ formulation; the seventh is negative control.

Chromatographic conditions：the column of Chromatographic：Kromasil C_18_(4.6 mm×250 mm,5 μm)；the mobile phase：Gradient elution was with methanol with 0.1% phosphoric acid-water，may you see in table 9；wavelength：210 nm；flow rate.：1ml /min；column temperature：30℃；injection volume 20μL。

Table 9 Chromatographic conditions

| min | A：methanol | B：0.1% Phosphoric acid water |
| --- | --- | --- |
| 0 | 20 | 80 |
| 3 | 22 | 78 |
| 10 | 24 | 76 |
| 20 | 25 | 75 |
| 30 | 27 | 73 |
| 45 | 40 | 60 |
| 60 | 45 | 55 |

***High-performance liquid chromatography(HPLC)***


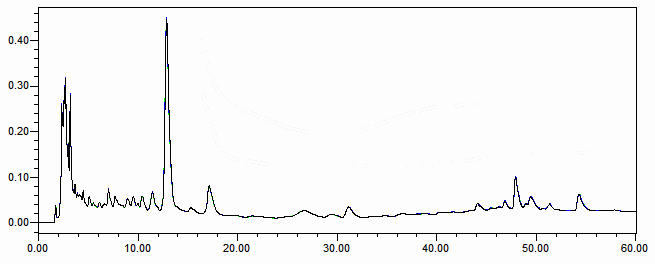


A

2

1

3

4


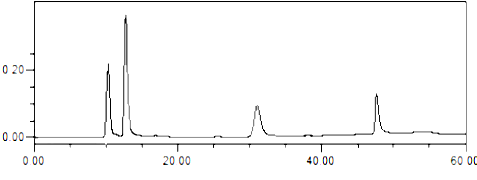
**Fig. 11:** (A )SEJZ chromatogram; (B )chromatogram of mixed standard;

3

B

4

2

1

1Syringin;2chlorogenic acid;3Eleutheroside E；4Cynaroside

**Table 10 The precision**

| project | | | 1 | | 2 | | 3 | | 4 | | 5 | | 6 | | RSD  （%） | |  |  |
| --- | --- | --- | --- | --- | --- | --- | --- | --- | --- | --- | --- | --- | --- | --- | --- | --- | --- | --- |
| Syringaldazine | | | 8255641 | | 8215617 | | 8145389 | | 8235072 | | 8199735 | | 8222641 | | 0.46 | |  |  |
| Chlorogenic acid | | | 12074298 | | 11996864 | | 11798367 | | 11967527 | | 12038719 | | 11899376 | | 0.84 | |  |  |
| Ciwujianoside E | | | 10931912 | | 10922437 | | 10854946 | | 10732451 | | 10823195 | | 10869395 | | 0.67 | |  |  |
| Wogonoside | | | 8526072 | | 8485578 | | 8385896 | | 8437501 | | 8482492 | | 8503379 | | 0.60 | |  |  |
|  |  | | | |  | |  | | **Table 11** | | **Stability** | |  | |  | |  |  |
| project | | | 0h | | 3h | | 6h | | 9h | | 18h | | 21h | | 24 h | | RSD  （%） | |
| Syringaldazine | | | 1571720 | | 1573137 | | 1587057 | | 1587425 | | 1651239 | | 1588400 | | 159960 | | 1.87 | |
| Chlorogenic acid | | | 14796754 | | 14765407 | | 14823693 | | 14787368 | | 15557636 | | 14848550 | | 14764452 | | 1.94 | |
| Ciwujianoside E | | | 1058710 | | 1057538 | | 1073931 | | 1050448 | | 1073617 | | 1075157 | | 1069210 | | 0.92 | |
| Wogonoside | | | 2150912 | | 2176512 | | 2255620 | | 2234854 | | 2252192 | | 2162694 | | 2029936 | | 3.63 | |
|  | |  | | |  | |  | |  | |  | |  | |  | |  |  |

**Table 12 Repeatability**

|  | 1 | 2 | 3 | content（%） | RSD（%） |
| --- | --- | --- | --- | --- | --- |
| Syringaldazine | 0.07 | 0.07 | 0.07 | 0.07 | 1.17 |
| Chlorogenic acid | 0.6 | 0.6 | 0.6 | 0.60 | 0.71 |
| Ciwujianoside E | 0.02 | 0.02 | 0.02 | 0.02 | 1.28 |
| Wogonoside | 0.04 | 0.04 | 0.04 | 0.04 | 1.04 |

**Table 13 The recovery rate of Syringaldazine**

| sample content  (mg) | addition  (mg) | measured amount（mg） | recovery  (%) | mean % | RSD  % |
| --- | --- | --- | --- | --- | --- |
| 0.36 | 0.18 | 0.54 | 98.19 |  |  |
| 0.36 0.36 0.36  0.36 | 0.18 0.18 0.36  0.36 | 0.54 0.54 0.72  0.72 | 98.45 96.59 97.75  99.40 | 97.89 | 1.12 |
| 0.36 | 0.36 | 0.71 | 96.74 |  |  |
| 0.36 | 0.54 | 0.89 | 96.54 |  |  |
| 0.36 | 0.54 | 0.89 | 98.01 |  |  |
| 0.36 | 0.54 | 0.90 | 99.33 |  |  |

**Table 14 The recovery rate of Chlorogenic acid**

| sample content (mg) | addition  (mg) | measured amount  （mg） | recovery  (%) | mean % | RSD  % |
| --- | --- | --- | --- | --- | --- |
| 3.02 | 1.50 | 4.50 | 98.45 |  |  |
| 3.02 3.02 3.02  3.02 | 1.50  3.00  3.00  3.00 | 4.51 4.48 6.01  5.92 | 98.91 97.25 99.47  96.49 | 98.23 | 1.08 |
| 3.02 | 3.00 | 5.98 | 98.56 |  |  |
| 3.02 | 4.50 | 7.42 | 97.81 |  |  |
| 3.02 | 4.50 | 7.51 | 99.67 |  |  |
| 3.02 | 4.50 | 7.41 | 97.46 |  |  |

**Table 15 The recovery rate of Ciwujianoside E**

| sample content (mg) | addition  (mg) | measured amount（mg） | recovery  (%) | mean % | RSD  % |
| --- | --- | --- | --- | --- | --- |
| 0.09 | 0.05 | 0.13 | 99.49 |  |  |
| 0.09 0.09 0.09  0.09 | 0.05 0.05 0.09  0.09 | 0.13 0.13 0.18  0.18 | 101.67  102.44 98.79  101.71 | 100.57 | 1.45 |
| 0.09 | 0.09 | 0.18 | 99.69 |  |  |
| 0.09 | 0.14 | 0.22 | 100.15 |  |  |
| 0.09 | 0.14 | 0.23 | 102.27 |  |  |
| 0.09 | 0.14 | 0.22 | 98.91 |  |  |

**Table 16 The recovery rate of Wogonoside**

| sample content (mg) | addition  (mg) | measured amount（mg） | recovery  (%) | mean % | RSD  % |
| --- | --- | --- | --- | --- | --- |
| 0.22 0.22 | 0.12 0.12 | 0.34 0.34 | 99.09 97.87 |  |  |
| 0.22 | 0.12 | 0.34 | 99.83 |  |  |
| 0.22 | 0.23 | 0.45 | 98.32 |  |  |
| 0.22 | 0.23 | 0.46 | 99.65 | 98.95 | 0.63 |
| 0.22 | 0.23 | 0.46 | 99.11 |  |  |
| 0.22 | 0.35 | 0.57 | 99.03 |  |  |
| 0.22 | 0.35 | 0.57 | 98.50 |  |  |
| 0.22 | 0.35 | 0.57 | 99.10 |  |  |

**Table 17 Determination of sample content**

| project | 1 | 2 | 3 | mean | content % |
| --- | --- | --- | --- | --- | --- |
| syringaldazine | 1623776 | 1625739 | 1571720 | 1607078 | 0.07 |
| Chlorogenic acid | 15023424 | 15060826 | 14796754 | 14960335 | 0.59 |
| Ciwujianoside E | 1076421 | 1061242 | 1058710 | 1065458 | 0.02 |
| wogonoside | 2188822 | 2164534 | 2150912 | 2168089 | 0.04 |

**Validation of Apigenin, Kaempferol, and Quercetin**

Chromatographic conditions：the column of Chromatographic：Kromasil C_18_(4.6 mm×250 mm,5 μm)；the mobile phase：Gradient elution was with acetonitrile with 0.02% phosphoric acid-water，may you see in table 18；wavelength：258 nm；flow rate.：1ml /min；column temperature：43℃；injection volume 10μL。

Table 18 Chromatographic conditions

| min | A：acetonitrile | B：0.02% Phosphoric acid water |
| --- | --- | --- |
| 0 | 73 | 27 |
| 20 | 63 | 37 |
| 40 | 45 | 55 |
| 50 | 45 | 55 |


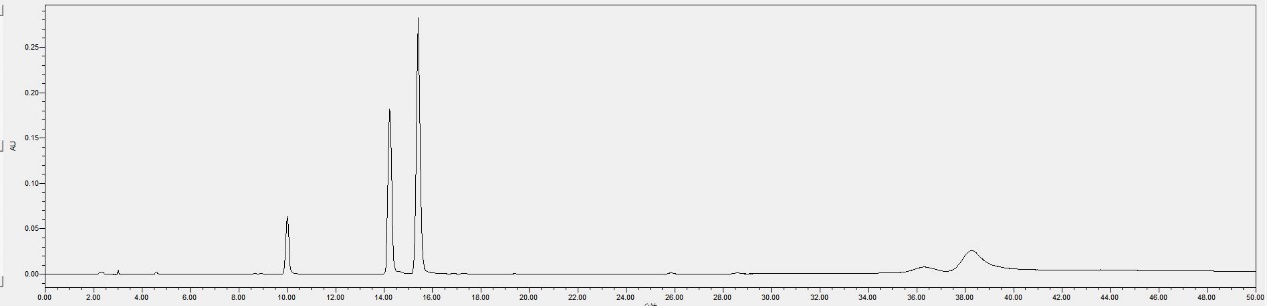


A A

A A

A A

A A

12 A

A A

3 A

A A

2 A

A A


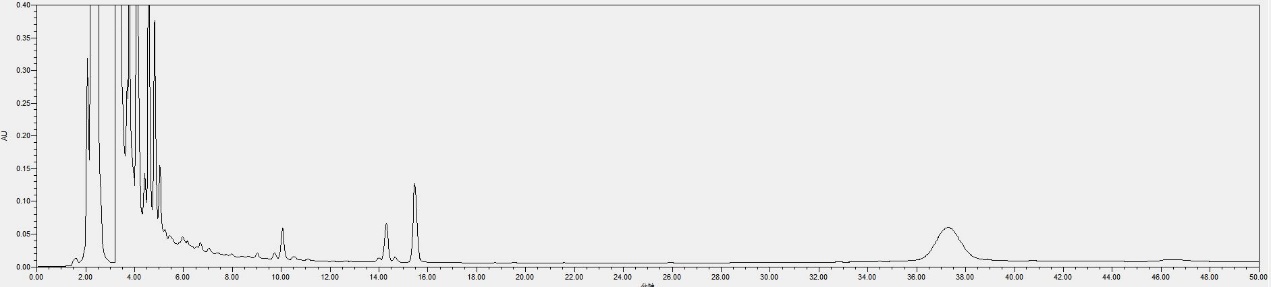


3 A

A A

2 A

A A

1 A

A A

B A

A A

**Fig. 11:** (A )SEJZ chromatogram; (B )chromatogram of mixed standard;

1 quercetin; 2 apigenin; 3 kaempferol

**Drug extract ratio and similar basic quality parameters**

Table 19 Compound extract ratio

|  | Acanthopanax  Senticosus  (g) | Lonicera japonica Thunb  (g) | Crataegus  pinnatifida Bunge  (g) | Auricularia auricula  (g) | TOTAL  (g) | EXTRACTION  (g) | DRUG EXTRACT RATIO (%) | MEAN RATIO  (%) |  |
| --- | --- | --- | --- | --- | --- | --- | --- | --- | --- |
| 1 | 265 | 177.5 | 177.5 | 133 | 753 | 236.1 | 31.35 |  |  |
| 2 | 177 | 118 | 118 | 88 | 501 | 164.0 | 32.73 | 31.89 |  |
| 3 | 177 | 118 | 118 | 88 | 501 | 158.2 | 31.58 |  |  |
